# Necessary and sufficient factors of keratinocytes in psoriatic dermatitis

**DOI:** 10.3389/fimmu.2024.1326219

**Published:** 2024-01-19

**Authors:** Teruki Dainichi, Reiko Matsumoto, Kenji Sakurai, Kenji Kabashima

**Affiliations:** ^1^ Department of Dermatology, Kagawa University Faculty of Medicine, Miki-cho, Japan; ^2^ Department of Dermatology, Kyoto University Graduate School of Medicine, Kyoto, Japan; ^3^ Agency for Science, Technology and Research (A*STAR) Skin Research Laboratories (ASRL), A*STAR, Singapore, Singapore

**Keywords:** psoriasis, TRAF6, p38, MAPK, CXCL2, HB-EGF, keratinocyte

## Introduction

Transcriptional activation of keratinocytes plays essential roles for the specific types of immune response of the skin (1). Psoriasis is a common chronic inflammatory skin disease that develops in middle-aged individuals with genetic predisposition (2). Clinical studies have demonstrated that tumor necrosis factor (TNF), interleukin (IL)-23, and IL-17 play pivotal roles in the pathogenesis of psoriasis. In addition, genome-wide association studies (GWASs) in patients with psoriasis have demonstrated that a genetic predisposition to T helper (T_H_) 17 responses and dysregulation of inflammatory signaling in immune and non-immune components, such as keratinocytes, contribute to the development of psoriasis. In particular, the identification of gene mutations in familial-type psoriasis suggests a unique role for keratinocyte signaling (2). However, the necessary and sufficient conditions for keratinocytes to develop psoriasis have not yet been identified.

Various animal models of psoriasis have been proposed. Among them, psoriatic dermatitis induced by daily epicutaneous application of imiquimod, a Toll-like receptor (TLR) 7/8 ligand, to mice is one of the most widely-used models. It reproduces several aspects of psoriasis including acanthosis and parakeratosis, neutrophil infiltration in the epidermis, and activation of the IL-23–IL-17 axis resulting proliferation of IL-17A-producing CD4^+^ T cells in the lesional skin (3). In addition, we have developed a new animal model for psoriasis by daily epicutaneous application with anisomycin, a p38-MAP kinase activator (4). It also reproduces histopathological and immunological aspects of psoriasis as well as imiquimod-induced dermatitis. Transcriptome analysis of the lesional skin revealed that approximately two thirds of the genes upregulated in imiquimod-induced dermatitis were also induced in anisomycin-induced dermatitis whereas some genes, such as *Defb3*, *S100a8/a9*, and *Il19*, were dominant in the imiquimod-induced dermatitis and others including *Defb4*, *Mmp13*, and *Il24* were dominant in the anisomycin-induced dermatitis (4).

Can we predict the necessary and sufficient conditions in keratinocytes by extracting response genes that are shared between each of the necessary and sufficient conditions for psoriatic dermatitis?

## A necessary condition — the TRAF6 pathway

TNF receptor associated factor 6 (TRAF6) is an intermediate signaling molecule between various types of receptors for exogenous or endogenous mediators and activation of nuclear factor-kappa B (NF-κB) and mitogen-activated protein kinase (MAP kinase) pathways (5). We previously demonstrated that mice lacking TRAF6, specifically in keratinocytes, are resistant to psoriatic inflammation induced by imiquimod (6).

## A sufficient condition — the p38-MAP kinase pathway

Then, which of the TRAF6 pathways, NF-κB pathways or MAP kinase pathways, is essential for the development and persistence of psoriasis? Notably, epidermis-specific depletion of the canonical NF-κB pathway using *Krt14*-Cre-mediated deletion of p65 (RelA) and c-Rel in keratinocytes leads to psoriatic dermatitis after birth (7). In contrast, we have shown that daily topical treatment of mouse ear skin with anisomycin is sufficient to induce psoriatic dermatitis in an IL-17-dependent manner (4).

Therefore, TRAF6-dependent p38-MAP kinase-mediated transcriptional activation of keratinocytes is expected to generate the necessary and sufficient conditions for psoriasis.

## Necessary and sufficient factors of keratinocytes in psoriatic dermatitis

The differentially expressed genes (DEGs) shared in the two conditions — (A) psoriatic dermatitis genes *in vivo* and (B) TRAF6–p38-response genes in keratinocytes — would embody the necessary and sufficient conditions for keratinocytes in psoriatic dermatitis. Venn diagrams using microarray data deposited in the Gene Expression Omnibus (GEO) database (accession numbers: GSE 30999, GSE101077, and GSE110658) (4, 6, 8) revealed the gene sets for each group. Group (A) included fifteen genes that were shared between dermatitis induced by imiquimod (84 genes) or anisomycin (306 genes) and psoriasis (518 genes), which were four times or more higher in lesional skin than in non-lesional skin. Group (B) included nine genes that were shared between imiquimod-induced genes fully abrogated in keratinocyte-specific TRAF6-deficient mice (80 genes), anisomycin-induced genes fully abolished by treatment with the p38 inhibitor BIRB796 (282 genes), and p38-response genes two times or more higher in primary-cultured keratinocytes after treatment with anisomycin (124 genes).

**Figure 1:**
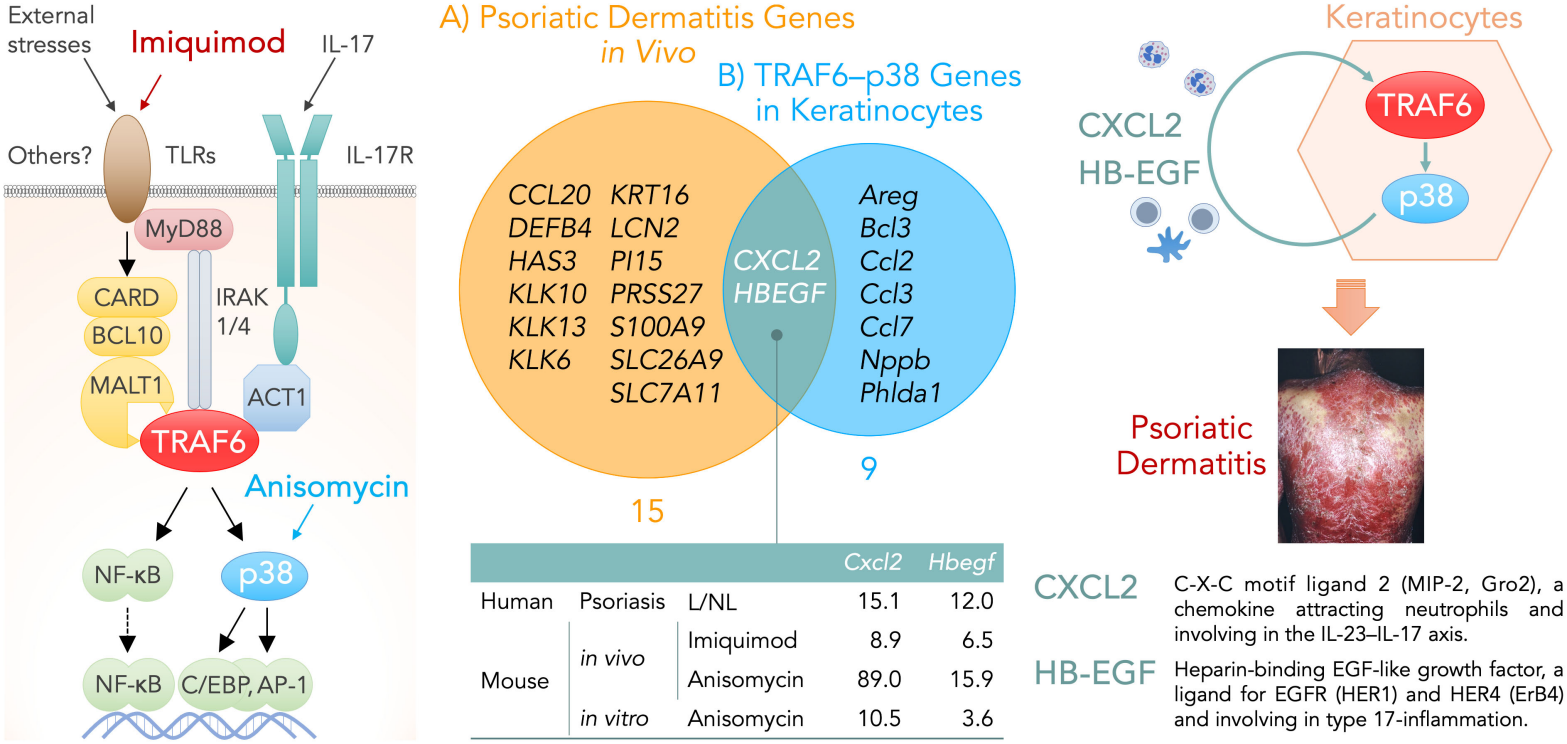
***Left***, keratinocyte signaling pathways that are involved in the development of psoriatic dermatitis. Imiquimod binds to TLR7/8 and triggers the activation of MyD88-dependent TLR signaling pathway. External stresses promote the formation of the CARD–BCL10–MALT1 (CBM) complex and subsequent activation of the TRAF6 pathway. *CARD14* is selectively expressed in the skin and its gain-of-function mutation are found in patients with familial psoriasis although the upstream molecule of CARD14 remains unknown. Anisomycin is a p38-MAP kinase activator. ***Middle***, Venn diagrams showing genes expressed by keratinocytes under the necessary and sufficient conditions for psoriatic dermatitis: A) Psoriatic dermatitis genes *in vivo* (fifteen genes), which were shared between psoriasis and its animal models induced by imiquimod or anisomycin, were four times or more higher in lesional skin than in non-lesional skin. B) TRAF6–p38-response genes in keratinocytes (nine genes), which were shared between imiquimod-induced genes fully abrogated in keratinocyte-specific TRAF6-deficient mice, anisomycin-induced genes fully abolished by treatment with the p38 inhibitor BIRB796, and p38-response genes two times or more higher in primary-cultured keratinocytes after treatment with anisomycin. The table indicates the number of fold-increases in the gene expression levels of *Cxcl2* and *Hbegf* under each condition. ***Right***, the production of CXCL2 and HB-EGF may represent necessary and sufficient conditions for keratinocytes in the TRAF6–p38 pathway-mediated inflammatory loop of psoriatic dermatitis. ACT1, NF-κB activator 1; AP-1, activator protein 1; BCL10, B-cell lymphoma/leukemia 10; CARD, caspase recruitment domain-containing protein; C/EBP, CCAAT/enhancer binding protein; CXCL2, C-X-C motif ligand 2; HB-EGF, heparin-binding epidermal growth factor-like growth factor; HER, human epidermal growth factor receptor; IL, interleukin; IL-17R, interleukin 17 receptor; IRAK, interleukin-1 receptor-associated kinase; L, lesional skin; MALT1, mucosa associated lymphoid tissue lymphoma translocation gene 1; MAP kinase, mitogen-activated protein kinase; MyD88, myeloid differentiation primary response protein 88; NF-κB, nuclear factor kappa B; NL, nonlesional skin; TLR, Toll-like receptor; TRAF6, tumor necrosis factor receptor-associated factor 6.

Venn diagrams for groups (A) and (B) demonstrated that only two genes, *CXCL2* and *HBEGF*, were common to psoriatic dermatitis genes *in vivo* and TRAF6–p38-response genes in keratinocytes (**Figure 1**).

## Discussion

The condition of keratinocytes for the transcription of these two genes, *CXCL2* and *HBEGF*, in keratinocytes may contribute to the necessary and sufficient conditions for triggering inflammatory loops in the epithelial-immune microenvironment (EIME) (2) in psoriasis.

*CXCL2* encodes C-X-C motif chemokine ligand 2 (CXCL2, also known as GRO2 or MIP-2), an 8 kDa-chemokine produced by activated monocytes and neutrophils. CXCL2 is expressed at sites of inflammation and recruits inflammatory cells, such as neutrophils, via its receptor C-X-C motif chemokine receptor 2 (CXCR2). CXCL2 gene expression is NF-κB-dependent, and the enzymatic activities of both coactivator-associated arginine methyltransferase (CARM1/PRMT4) and transcriptional coactivator p300/CREB-binding protein are necessary for the NF-κB-mediated transactivation (9). p38-MAP kinase pathway is involved in the stabilization and translation of CXCL2 mRNAs (10, 11). Mechanistically, MAP kinase-activated protein kinase 2 (MK2), a downstream kinase of p38, phosphorylates heterogeneous nuclear ribonucleoprotein (hnRNP) A0, which binds to AU-rich elements (AREs) within the 3’-untranslated regions of CXCL2 mRNAs and stimulates their stabilization and translation (5, 11).

Epidermal keratinocytes produce chemoattractants, including CXCL2, and recruit neutrophils to the skin lesions of patients with psoriasis and its animal models (12). IL-17 stimulates the production of multiple chemokines, including CXCL2, in multiple cell lineages (13). In an acute trinitrobenzene sulfonic acid (TNBS)-induced colitis model, the production of CXCL2 in the colon and its severity are reduced in *Il17ra* ^–/–^ mice (14). Imiquimod-induced psoriatic dermatitis triggers the transcriptional expression of CXCL2 and CXCR2 in the earliest phase and is blunted by treatment with a selective CXCR2 antagonist (15). In contrast, evidence from other diseases has suggested the involvement of CXCL2 in the induction of the IL-23–IL-17 axis and the type 17 inflammatory loop. In patients with inflammatory bowel diseases, CXCR1^+^ CXCR2^+^ neutrophils that infiltrate the colon are the main sources of IL-23 (16). In addition, the antibody blockade of CXCR2 signaling reduces the expression levels of tissue IL-23 in the liver and intestine at the initial stage of graft-versus-host disease (GVHD) and attenuates disease severity (17). Furthermore, photodynamic therapy in BALB/c Colo26-HA tumor-bearing mice rapidly induces the accumulation of T_H_17 cells in tumor-draining lymph nodes (TDLNs), and IL-17 promotes neutrophil migration into TDLNs across HEVs through preferential interactions between CXCR2 and CXCL2, but not CXCL1 (18). Moreover, the development of Candida-induced keratitis is blocked by anti-IL-17A or anti-IL-23p19 antibodies and is defective in nude mice. However, CXCL2 is sufficient to restore Candida keratitis in nude mice (19). Therefore, it is likely that the CXCL2–IL-23–IL-17 loop in the EIME drives type 17 inflammation in patients with psoriasis.

*HBEGF* encodes a transmembrane protein called pro-heparin-binding epidermal growth factor-like growth factor (HB-EGF). The processing of pro-HB-EGF via ectodomain shedding by proteases, including a disintegrin and metalloproteinase (ADAM)17/TNF-α converting enzyme (TACE) and matrix metalloproteinases (MMPs), produces mature HB-EGF, a member of the EGF protein family (20). It is worth noting that the expression levels of several genes coding ADAMs and MMPs are significantly higher in the psoriasis lesional skin than in the nonlesional skin (8). HB-EGF is a ligand of epidermal growth factor receptor (EGFR)/human EGFR (HER)1 and HER4/ErbB4, whose activation triggers a series of signaling cascades and results in a variety of effects, including cell proliferation and migration. In contrast, a release of ATP in a challenging environment induces HB-EGF synthesis and release through the p38-MAP kinase pathway in human keratinocytes (21).

HB-EGF is involved in the development of pathological conditions associated with type 17 inflammation. T_H_17-induced airway remodeling in a mouse asthma model leads to the epithelial overexpression of HB-EGF and is abolished by HB-EGF blockade (22). Transgenic mice that express HB-EGF throughout the intestine develop serrated polyps, in which the frequency of IL-17-producing γδ T cells is higher than in unaffected surrounding tissue or the wild-type (23).

HB-EGF is located in the basal layer of healthy and nonlesional skin, overexpressed in the suprabasal layers of uninvolved skin and marginal lesions in psoriasis, but not in the center part of psoriatic lesions (24). In human keratinocytes, HB-EGF and IL-17A synergistically induce IκBζ expression, which is essential for the induction of psoriasis signature genes *in vitro*, in a p38-MAP kinase-dependent manner (25). Notably, mice lacking HB-EGF specifically in keratinocytes show defects in wound healing (26), a process that simulates psoriatic conditions. EGFR is expressed in the basal and suprabasal layers of normal epidermis and is highly expressed throughout the epidermis in psoriatic lesions (27). Of note, there are reported cases treated for cancers with EGFR-blockade agents (cetuximab and erlotinib) resulting resolution of concomitant psoriasis (28). Expression levels of HER4 in epidermal keratinocytes are higher in the psoriasis lesional skin than those from normal skin (29). HER4 is also expressed in CD4^+^ T cells from patients with psoriasis, and treatment with HER4 siRNA reduced mouse IL-17A^+^ CD4^+^ T cells *in vitro* and imiquimod-induced dermatitis *in vivo* (30). These results suggest that HB-EGF is a critical downstream mediator of the TRAF6–p38 pathway in psoriatic dermatitis.

In conclusion, our previous findings suggest that TRAF6 in keratinocytes is a necessary factor and cutaneous activation of p38-MAP kinase is a sufficient factor for psoriatic dermatitis. Venn diagrams of their transcriptome data show that CXCL2 and HB-EGF are the only two factors produced by keratinocytes under the necessary and sufficient conditions for psoriatic dermatitis. This interpretation allows us to propose novel drug targets for psoriasis.

## Author contributions

TD: Conceptualization, Formal analysis, Supervision, Writing – original draft, Writing – review & editing. RM: Data curation, Formal analysis, Investigation, Writing – review & editing. KS: Data curation, Formal analysis, Investigation, Writing – review & editing. KK: Resources, Writing – review & editing.
